# Supplementary material for: The Evolution and Characterization of the RNA Interference Pathways in Lophotrochozoa
Source: Genome Biol Evol. 2024 May 7;16(5):evae098. doi: 10.1093/gbe/evae098 (PMC11114477; doi:10.1093/gbe/evae098)
Supplement: evae098_Supplementary_Data [file evae098_supplementary_data.zip › Supplementary_materials.zip!/selected_miRNAs/NoGe_selected_miRNAs/mirdeep_pdf_files/NMRB01000076.1_Notospermus_geniculatus_strain_Ushimado_scaffold76,_whole_genome_shotgun_sequence_2794.pdf]

Provisional ID	:	NMRB01000076.1_Notospermus_geniculatus_strain_Ushimado_scaffold76,_whole_genome_shotgun_sequence_2794
Score total	:	482.6
Score for star read(s)	:	3.9
Score for read counts	:	477.3
Score for mfe	:	2
Score for randfold	:	0
Score for cons. seed	:	-0.6
Total read count	:	948
Mature read count	:	886
Loop read count	:	0
Star read count	:	62

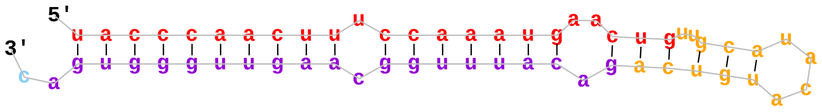

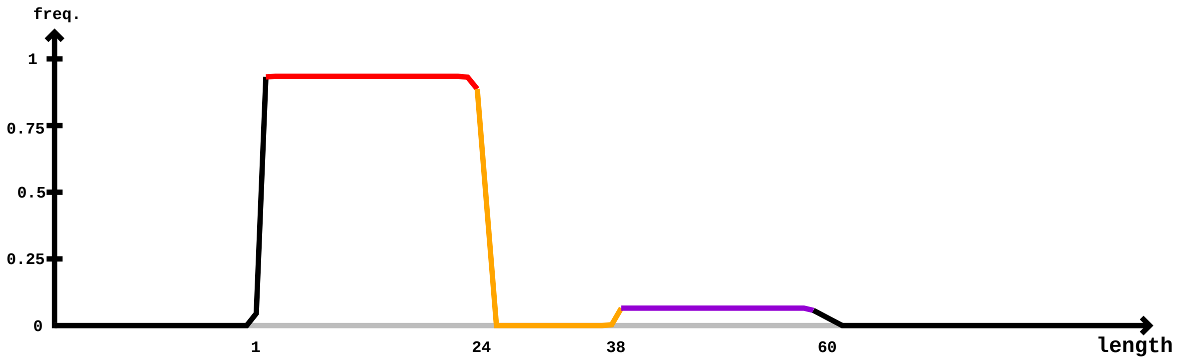

Mature Star

```
5'- ucacauuugccuagagcaguuacccaacuuuccaaaugaacuguugcauacaugucagacauuuggcaaguuggugacuguuccaggauugaaauuucugacaguaggggc -3'   obs
    ucacauuugccuagagcaguuacccaacuuuccaaaugaacuguugcauacaugucagacauuuggcaaguuggugacuguuccaggauugaaauuucugacaguaggggc        exp
    ........((((.(((((((((((((((((.(((((((..(((..(((....)))))).))))))).)))))))))))))))))(((((.......))))).......))))   reads   mm   sample
    ....................uacccaacuuuccaaaugaacu......................................................................   6       0    Mm1
    ....................uacccaacuuucUaaaugaacug.....................................................................   1       1    Mm1
    ....................uacccaacuuuccaaaCgaacug.....................................................................   1       1    Mm1
    ....................uacAcaacuuuccaaaugaacug.....................................................................   1       1    Mm1
    ....................uacccaacuuuccaaaugaacug.....................................................................   88      0    Mm1
    .........................................................gacauuuggcaaguugAguga..................................   1       1    Mm1
    .........................................................gacauuuggcaaguuggguga..................................   10      0    Mm1

    ...................uuacccaacuuuccaaaugaacu......................................................................   2       0    Mm2
    ...................uuacccaacuuuccaaaugaacug.....................................................................   4       0    Mm2
    ...................uuacccaacuuuccaaUugaacug.....................................................................   1       1    Mm2
    ...................uuacccaacuuuccaaauCaacug.....................................................................   1       1    Mm2
    ....................uacccaacuuuccaaaugaac.......................................................................   1       0    Mm2
    ....................uacccaacuuuccaaauCaac.......................................................................   1       1    Mm2
    ....................uacccaacuuuccaaaugaacu......................................................................   14      0    Mm2
    ....................uacccaacuuuccaaauCaacu......................................................................   7       1    Mm2
    ....................uacccaacuuuccaaauCaacug.....................................................................   3       1    Mm2
    ....................uacccaacuuucUaaaugaacug.....................................................................   1       1    Mm2
    ....................uacccaacuuuccaaauAaacug.....................................................................   2       1    Mm2
    ....................uacccaacuuuccaaaugaacug.....................................................................   352     0    Mm2
    ....................uacccaacuuuccaaaAgaacug.....................................................................   1       1    Mm2
    ....................uacccaacGuuccaaaugaacug.....................................................................   1       1    Mm2
    ....................uacccGacuuuccaaaugaacug.....................................................................   1       1    Mm2
    ....................uacccaacuuuccaGaugaacug.....................................................................   1       1    Mm2
    ....................uacccaacuuuccaaaugUacug.....................................................................   1       1    Mm2
    ....................uUcccaacuuuccaaaugaacug.....................................................................   1       1    Mm2
    ....................uacccaacuuuUcaaaugaacug.....................................................................   1       1    Mm2
    ....................uacccaacuuGccaaaugaacug.....................................................................   1       1    Mm2
    ....................uGcccaacuuuccaaaugaacug.....................................................................   3       1    Mm2
    ....................uacccaacuuuccaaauUaacug.....................................................................   2       1    Mm2
    ....................uaccAaacuuuccaaaugaacug.....................................................................   2       1    Mm2
    ....................uacccaacuuuccaaaCgaacug.....................................................................   4       1    Mm2
    ....................uacccaacuuuccaaaugaacugu....................................................................   2       0    Mm2
```

```
                    Mature                                Star
ucacauuugccuagagcaguuacccaacuuuccaaaugaacuguugcauacaugucagacauuuggcaaguuggguga cuguuccaggauugaaauuucugacaguaggggc
.....................acccaacuuuccaaaugaacug.....................................................................  1    0   Mm2
........................................................agacauuuggcaaguugggugU..................................  1    1   Mm2
.........................................................gacauuuggcaaguuggguga..................................  3    0   Mm2
.........................................................gacauuuggcaaguugggugac.................................  1    0   Mm2
.........................................................gacauuuggcaaguugggugacA................................  1    1   Mm2
.........................................................gacauuuggcaaguugggugacu................................  3    0   Mm2

...................uuacccaacuuuccaaaugaacug.....................................................................  15   0   Fm2
....................uacccaacuuuccaaauAaacug.....................................................................  1    1   Fm2
....................uacccaacuuuccaaauCaacug.....................................................................  1    1   Fm2
....................uacccaacuuuccaaaugaacuU.....................................................................  1    1   Fm2
....................uacccaacuuuccaaaugaacug.....................................................................  29   0   Fm2
....................uGcccaacuuuccaaaugaacug.....................................................................  1    1   Fm2
....................uacccaacuuuccaaaGgaacug.....................................................................  1    1   Fm2
.........................................................gacauuuggcaaguugggug...................................  7    0   Fm2

....................uacccaacuuuccaaaugaacug.....................................................................  22   0   Fm1
.........................................................gacauuuggcaaguuggguga..................................  4    0   Fm1

...................uuacccaacuuuccaaaugaacu......................................................................  1    0   Mm3
...................uuacccaacuuuccaaaugaacug.....................................................................  8    0   Mm3
....................uacccaacuuuccaaaugaacu......................................................................  4    0   Mm3
....................uacccaacuuAccaaaugaacug.....................................................................  1    1   Mm3
....................uacccaacuuuccaaaugaacug.....................................................................  29   0   Mm3
....................Cacccaacuuuccaaaugaacug.....................................................................  1    1   Mm3
....................uacccaacuuuccaaaugaacugu....................................................................  1    0   Mm3
.........................................................gacauuuggcaaguugggug...................................  1    0   Mm3
.........................................................gacauuuggcaGguugggugac.................................  1    1   Mm3
.........................................................gacauuuggcaaguugggugac.................................  1    0   Mm3
.........................................................gacauuuggcaaguugggugacG................................  1    1   Mm3
.........................................................gacauuuggcaaguugggugacu................................  3    0   Mm3

...................uuacccaacuuuccaaaAgaacug.....................................................................  1    1   Fm3
...................uuacccaacuuuccaaaugaacug.....................................................................  9    0   Fm3
...................uuacccaacuuuccaaaCgaacug.....................................................................  1    1   Fm3
....................uacccaacuuuccaaaugaac.......................................................................  1    0   Fm3
....................uacccaacuuuccaaaugaacu......................................................................  8    0   Fm3
....................uaAccaacuuuccaaaugaacug.....................................................................  1    1   Fm3
....................uacccaacuuuccaaaGgaacug.....................................................................  1    1   Fm3
....................uacccaacCuuccaaaugaacug.....................................................................  1    1   Fm3
....................uacccaacuuuccaaaugaacug.....................................................................  231  0   Fm3
....................uacccGacuuuccaaaugaacug.....................................................................  1    1   Fm3
....................uUccaacuuuccaaaugaacug......................................................................  1    1   Fm3
....................uacccaacuuuccaaauCaacug.....................................................................  1    1   Fm3
....................uacccaacuuuccaaaugaacugu....................................................................  4    0   Fm3
.....................acccaacuuuccaaaugaacug.....................................................................  1    0   Fm3
........................................................agacauuuggcaaguuggguga..................................  2    0   Fm3
.........................................................gacauuuggcaaguuggguga..................................  3    0   Fm3
.........................................................gacauuuggcaaguugggugU..................................  1    1   Fm3
.........................................................gacauuuggcaaguugggugac.................................  10   0   Fm3
.........................................................gacauuuggcaaguugggugaA.................................  1    1   Fm3
.........................................................gacauuuggcaaguugggugacu................................  5    0   Fm3
.........................................................gacauuuggcaaguugggugacA................................  2    1   Fm3
```
